# Fitness of unregulated human Ras mutants modeled by implementing computational mutagenesis and machine learning techniques

**DOI:** 10.1016/j.heliyon.2019.e01884

**Published:** 2019-06-12

**Authors:** Majid Masso, Arnav Bansal, Preethi Prem, Akhil Gajjala, Iosif I. Vaisman

**Affiliations:** School of Systems Biology, George Mason University, 10900 University Blvd. MS 5B3, Manassas, Virginia, 20110, USA

**Keywords:** Bioinformatics, Biophysics, Cancer research, Computational biology, Systems biology

## Abstract

Ras proteins play a pivotal role as oncogenes by participating in diverse signaling events, including those linked to cell growth, differentiation, and proliferation. Using experimental fitness data and implementing artificial intelligence and a computational mutagenesis technique, we developed models that reliably predict fitness for all single residue mutants of H-*ras* proto-oncogene protein p21. The computational mutagenesis generated a feature vector of protein structural changes for each variant, and these data correlated well with fitness. Random forest classification and tree regression machine learning algorithms were implemented for training predictive models. Cross-validations were used to evaluate model performance, and control experiments were performed to assess statistical significance. Classification models revealed a balanced accuracy rate as high as 82%, with a Matthew's correlation of 0.63, and an area under ROC curve of 0.90. Similarly, regression models displayed Pearson's correlation reaching 0.79. On the other hand, control data sets led to performance values consistent with random guessing. Comparisons with several related state-of-the-art methods reflected favorably on our trained models. This H-Ras proof-of-principle study suggests a complementary approach for understanding mechanisms with which other proteins are involved in oncogenesis, including related Ras isoforms, and for providing useful insights into designing future diagnostic and treatment modalities.

## 1 Introduction

Among the class of small GTPase molecules driving cell signaling pathways in the cellular architecture of all organisms, the p21 Ras proteins provide an essential role with respect to cellular proliferation, differentiation, and apoptosis (Pai et al., 1990; Rosnizeck et al., 2010). The structurally related human Ras isoforms (N– K- and H-Ras) obtained through alternative splicing events are the products of proto-oncogenes found in up to 30% of human tumors, with rates reaching as high as 90% for pancreatic cancer (Coppe et al., 2018; Mattingly, 2013; Mo et al., 2018; Rosnizeck et al., 2010; Simanshu et al., 2017). Wild type Ras acts as a molecular switch, cycling between the active (ON) and inactive (OFF) conformational states when complexed to guanosine triphosphate (GTP) and guanosine diphosphate (GDP), respectively (Pai et al., 1990; Simanshu et al., 2017). Ras in the ON conformation transduces downstream signals by interacting with effector proteins (e.g., Raf kinase), and although intrinsic GTPase activity of Ras is low, it is catalyzed by the binding of a GTPase activating protein (GAP) (Bandaru et al., 2017; Pai et al., 1990; Simanshu et al., 2017). Similarly, intrinsic GDP release in the OFF state is slow until Ras is bound by a guanine nucleotide exchange factor (GEF). Mutant Ras proteins isolated from human tumors are found to remain trapped in the active conformation bound to GTP due to the inability of GAP to recycle them quickly enough to the inactive GDP bound form (Pai et al., 1990; Rosnizeck et al., 2010).

In a recent study (Bandaru et al., 2017), saturation point-mutagenesis was performed on human H-Ras to create libraries such that each amino acid position was individually randomized. Next, the libraries were cloned into an *E. coli* bacterial two-hybrid expression vector and deep-sequenced to ensure a uniform distribution of Ras variants. Lastly, the effects of amino acid residue point mutations on Ras were determined by transforming *E. coli* cells with the libraries, screening with the two-hybrid system, and deep-sequencing both before and after selection. The experimental data obtained were used to calculate fitness scores that quantified functional effects associated with all single residue Ras mutants relative to the native protein. Multiple experiments were performed on Ras mutants, corresponding to regulated (under the control of GAP and GEF), attenuated (GAP without GEF), and unregulated (neither GAP nor GEF) conditions. Tables containing fitness scores for all 19 single residue Ras mutants at each of positions 2–166 in the protein were provided with that manuscript as supplementary material (https://elifesciences.org/articles/27810/figures#SD2-data).

**Fig. 1:**
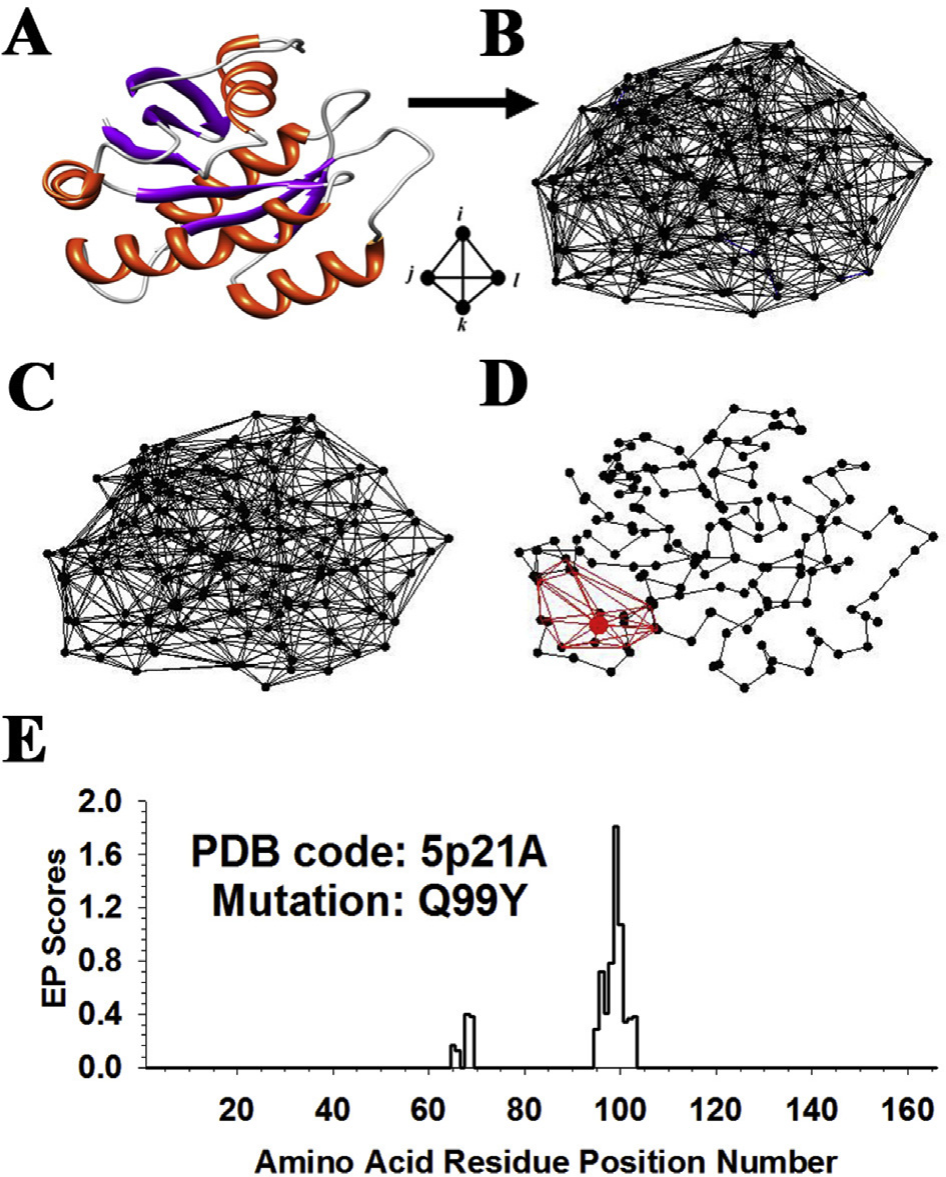
(A) Ribbon diagram of the human H-Ras protein based on the atomic coordinates in Protein Data Bank (PDB) accession code 5p21A (residues 1–166 of H-Ras in a complex with GNP, a slowly hydrolyzing GTP analogue). (B) Complete Delaunay tessellation of the H-Ras protein, generated using the residue C-alpha coordinates as tetrahedral vertices, and consisting of 900 tetrahedra. (C) After removing all edges longer than 12 Å, there remain 734 tetrahedra in the tessellation. (D) Amino acid position Q99 (larger red point) is shared as a vertex by a total of 18 tetrahedra (edges in red), and it has 12 nearest neighbors (i.e., positions that are connected to Q99 via a tessellation edge). (E) Visual display of the 166-dimensional (166D) residual profile vector for Ras variant Q99Y. The residual score of Q99Y is equivalent to the environmental perturbation (EP) score at position 99 in the plot.

To assess the structural impacts of these Ras variants, here we implemented a previously derived computational mutagenesis technique that employs a four-body statistical potential (Masso and Vaisman, 2008, 2010a) and the solved structure of a native Ras protein (Fig. 1A) (Pai et al., 1990). For any mutant defined by amino acid residue substitution(s) at one or more positions in the wild type protein, the methodology computes the overall change to protein sequence-structure compatibility (the mutant *residual score*) and quantifies environmental perturbation (EP) scores for all residue positions in the protein, with the latter forming a vector of EP scores (the mutant *residual profile*). Using this approach, residual scores and residual profile vectors were calculated for all 165 × 19 = 3135 Ras variants. The structure-function relationship inherent in Ras was elucidated by comparing the residual scores of these Ras variants with their experimental fitness data. The residual scores were also used to effectively cluster Ras residue positions according to both polarity as well as regulatory or functional roles in the protein. Given that the procedure utilizes an isolated Ras structure (i.e., no bound GAP or GEF) and is conducted under static conditions (i.e., temporal effects of GAP and GEF on Ras are not quantifiable), only the unregulated experimental fitness data were used in our study. Moreover, the residual profiles were used as inputs to train models for predicting Ras variant (unregulated) fitness values by implementing state-of-the-art machine learning algorithms. The models developed in this work outperformed several related methods and suggest an innovative approach for investigating the impact of mutations in other proto-oncogenic proteins.

## 2 Materials and methods

### 2.1 Four-body statistical potential

Underpinning the computational mutagenesis is a knowledge-based energy function generated from a set of 1417 diverse (<2.2 Å), high resolution (<30% sequence identity) X-ray protein structures (http://binf.gmu.edu/automute/tessellatable1417.txt) culled from the Protein Data Bank (PDB) (Berman et al., 2000) by using the PISCES server (Wang and Dunbrack, 2003). Structures were represented as discrete sets of points in three-dimensional (3D) space based on the C-alpha atomic coordinates of the constituent amino acid residues. Each protein structure point-set was supplied to the Qhull software (Barber et al., 1996) for generating its Delaunay tessellation (Fig. 1B), a well-established computational geometry algorithm (de Berg et al., 2008) that yields a 3D convex hull consisting of a packed tiling formed by hundreds of space-filling, non-overlapping, irregular tetrahedra whose vertices are the C-alpha coordinates of the residues. Tetrahedral simplices in a tessellation objectively identify at their vertices quadruplets of interacting residues in the structure; however, the convexity requirement occasionally leads to long edges that connect C-alpha coordinates of non-interacting surface residues. Therefore, all tetrahedral edges longer than 12 Å were immediately removed prior to further analysis, to ensure that the vertices of each remaining tetrahedral simplex correspond to a quadruplet of residues whose respective backbone and side-chain atoms are at a distance close enough to one another to form non-covalent interactions (Fig. 1C).

Any one of 8855 possible types of interacting residue quadruplets (*i*, *j*, *k*, *l*) may exist at the four vertices of a tetrahedral simplex in a protein structure tessellation, since the quadruplets are unordered (i.e., permutations are not counted) and residue types may occur repeatedly in any given quadruplet (Brualdi, 1999; Masso, 2015; Masso and Vaisman, 2014). For each type of quadruplet, a relative frequency of occurrence *f*_*ijkl*_ was calculated as the proportion of all tetrahedral simplices comprising the 1417 protein structure tessellations for which the quadruplet (*i*, *j*, *k*, *l*) appears at the four vertices. A rate expected by chance *p*_*ijkl*_ was also computed, first by determining the relative frequencies of occurrence for the 20 types of amino acids based on all residues that make up the 1417 protein structures, and then by applying a multinomial reference distribution:

$p_{ijkl}=\frac{4!}{\prod_{n=1}^{20}\left(t_{n}!\right)}\prod_{n=1}^{20}a_{n}^{t_{n}}$, where *a*_*n*_ represents the relative frequency of occurrence of residue type *n* such that $\sum_{n=1}^{20}a_{n}=1$, and *t*_*n*_ corresponds to the number of times that residue type *n* is repeated in the quadruplet (*i*, *j*, *k*, *l*) so that $\sum_{n=1}^{20}t_{n}=4$. Modeled after the inverted Boltzmann principle, an interaction energy score for each residue quadruplet (*i*, *j*, *k*, *l*) was computed as *s*_*ijkl*_ = log(*f*_*ijkl*_/*p*_*ijkl*_) (Sippl, 1993, 1995), and these scores make up the four-body potential (http://binf.gmu.edu/automute/potential_1417_cut12.txt).

The four-body potential can be used to calculate the *total potential* of any native protein with known 3D structure as follows: (1) tessellate the C-alpha coordinates of its constituent residues and remove edges longer than 12 Å; (2) score each tetrahedron in the tessellation based on the quadruplet of residues (*i*, *j*, *k*, *l*) represented on its four vertices as *s*_*ijkl*_ by using the four-body potential; (3) add up the scores of all the tetrahedra. A *residue environment score (RES)* can also be calculated for each amino acid position in the protein, by adding up the scores of all tetrahedra that share the C-alpha coordinate of the amino acid as a vertex; moreover, the ordered vector of collective RES scores for all amino acid positions in the protein defines a *3D-1D potential profile* (Bowie et al., 1991).

### 2.2 Computational mutagenesis

To compute the total potential and potential profile for any single residue mutant of the native protein, the same procedure outlined above can be used after making an initial adjustment to the tessellation. Tetrahedral scores and subsequent calculations for the mutant are determined once the amino acid type associated with the C-alpha coordinate of the position to be mutated in the tessellation is altered to reflect the replacement residue. The difference between the mutant and native protein total potentials is referred to as the *residual score* and quantifies the relative change in sequence-structure compatibility upon mutation (Masso, 2015; Masso and Vaisman, 2010b). Additionally, the difference between their potential profiles (component-wise subtraction of vectors) is termed a *residual profile*, whose components reflect *environmental perturbation (EP)* scores at all amino acid positions due to the mutation (Fig. 1D, E) (Masso, 2015; Masso and Vaisman, 2008, 2010b, 2014). A straightforward yet detailed geometrical argument shows that the residual score is precisely the EP score at the mutated position (Masso, 2015; Masso and Vaisman, 2010b). Finally, we define a *comprehensive mutational profile (CMP)* score at each protein position as the calculated average of the residual scores for all 19 amino acid substitutions, quantifying the mean impact to protein sequence-structure compatibility of replacing the native residue at that position (Masso et al., 2006).

### 2.3 Ras variant feature vectors, machine learning, and model evaluation

Using their 166-dimensional (166D) residual profiles, the Ras variants were presented as feature vectors to supervised machine learning algorithms for training predictive models of fitness based on two fundamental approaches. The term supervised signifies that the fitness data for the Ras variants in the training set are also provided to the algorithm. In the first case, three components (position number of the mutation, native and replacement residues) were appended to the left of the residual profiles and one component (fitness) to the right to yield 170D feature vectors for the Ras variants (http://binf.gmu.edu/automute/Ras_variant_residual_profiles.csv). The fitness component is the *output attribute* (i.e., dependent variable), while all preceding components are referred to as *input attributes* (i.e., independent variables or predictors). Each supervised learning algorithm uses the given training data (i.e., the Ras variants) to learn a predictive model, a complex non-linear function of the input attributes that yields the output attribute. The goal is a robust model capable of accurately predicting new data not used in the training set, one that is more likely achieved by using a large, diverse training set that is as uniformly distributed throughout the sample space as possible, as well as by avoiding over- and under-fitting of the model to the training data.

In the second approach (Masso, 2015), a 28D feature vector was generated for each Ras variant, with components that focus locally on the mutated position as well as its six nearest neighbors based on tetrahedral edge lengths (Euclidean distance) between pairs of C-alpha coordinates. By selecting six nearest neighbors, a compromise was achieved between characterizing more fully the mutated position neighborhood and eliminating additional mutants from the data set because they do not possess the requisite number of neighbors. The 27 input attributes for each Ras variant included: mutated position number, as well as the native and replacement amino acids; residual score (i.e., EP score at the mutated position obtained from the residual profile); EP scores at the six nearest neighbors (ordered by distance) obtained from the residual profile; amino acid identities at the six neighbors (ordered); difference in primary sequence numbers between that of the mutated position and those of the six neighbors (ordered); mean volume and mean tetrahedrality for all tetrahedra in the tessellation of Ras that share the C-alpha coordinate of the mutated residue as a vertex; tessellation-based location of the mutated position in the protein (surface, undersurface, or buried), and the number of edge contacts it has with surface residue positions; and the secondary structure at the mutated position. The Ras variant fitness output attribute provided the final (28th) feature vector component (http://binf.gmu.edu/automute/Ras_variant_local_profiles_classification.csv and http://binf.gmu.edu/automute/Ras_variant_local_profiles_regression.csv).

To train predictive models of Ras variant fitness and to evaluate their performance, tree regression (REPTree, reduced-error pruned tree) and random forest classification algorithms were implemented with the Weka software package of machine learning tools (Frank et al., 2004; Smith and Frank, 2016). Regression models predict the experimentally determined numerical fitness values, and those values are included as the output attribute for each Ras variant feature vector in the training data set. Classification algorithms require training set feature vectors to have categorical (i.e., increased versus decreased Ras variant fitness relative to the native protein) rather than real-valued output attributes, and the trained models predict the class membership. We used the median fitness value (-0.06) over all 3135 Ras variants to group them into the two equally-sized fitness categories. Additional details regarding the implementation of these methods (e.g., parameter values) are provided in the Results.

We applied both leave-one-out and tenfold cross-validation (LOOCV and 10-fold CV, respectively) procedures to evaluate the performance of the models. Using 10-fold CV, the Ras variants were initially placed into ten disjoint subsets of equal size, and the following iterative procedure was performed: (1) one subset was held-out (10% of the data), while a model was trained with the combined Ras variants from the other nine subsets (90% of the data); (2) the trained model was used for predicting the (known) Ras variants in the held-out set; (3) the procedure was repeated in order for each subset to be held-out once and predicted. With LOOCV, each Ras variant formed its own subset (i.e., a singleton) in the initial step, prior to running an iterative procedure identical to 10-fold CV. Categorizing the fitness of Ras variants as either increased (P, positive class) or decreased (N, negative class) relative to native Ras, random forest classifier predictions were evaluated by calculating Se = sensitivity = TP/(TP + FN), Sp = specificity = TN/(TN + FP), and PPV = positive predictive value (i.e., precision) = TP/(TP + FP). Performance measures that are robust to differences in category sizes were also computed, including balanced accuracy rate (BAR) = 0.5 × [Sensitivity + Specificity], Matthew's correlation coefficient (MCC), and area (AUC) under the receiver operating characteristic (ROC) curve. For tree regression predictions, Pearson's correlation coefficient (*r*) between the actual and predicted fitness values and the root mean squared error (RMSE) were reported. Additionally, all tree regression model fitness values (actual and predicted) were converted to categories by using median fitness as a threshold, in order to compute the classification performance measures given above and to subsequently compare the results with those of the random forest model.

All of the work presented here was performed using a laptop PC. Time required for generating all Ras variant residual profile vectors took on the order of a few hours. Similarly, each LOOCV testing run took a few hours, while each 10-fold CV run took several minutes. Lastly, predictions using a chosen model took less than 1 min.

## 3 Results and discussion

### 3.1 Ras structure-function relationships

**Fig. 2:**
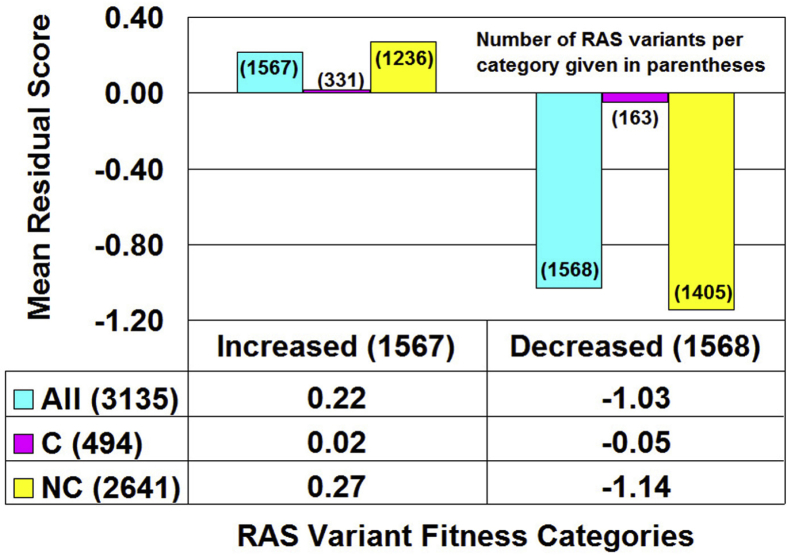
Ras protein structure-function relationship. All refers to the set of all 3135 Ras variants with experimental fitness data, and C/NC are the subsets of these variants that represent conservative/non-conservative amino acid substitutions of the native residue. The data in the table below the figure are means of the residual scores for the associated subsets of the mutants. All numbers in parentheses located either on the graph or in the table row/column headings are counts for the total number of mutants in that subset.

Residual scores were calculated for all 3135 Ras variants upon each of 19 possible amino acid residue replacements at positions 2–166 in the Ras structure (PDB accession code: 5p21) for which experimental fitness data relative to the native protein were available. Next, the mutants were grouped according to their fitness categories (i.e., increased versus decreased relative to the native Ras) and residual scores were averaged over each category (Fig. 2, black bar graph). A Ras structure (i.e., mean residual score) – function (i.e., fitness category) relationship is revealed by Fig. 2, whereby functional impairments of Ras mutants correlate with detrimental impacts to structure. Moreover, the difference between the mean residual scores for the two fitness categories is statistically significant (*t*-test: *p* < 0.0001).

The Ras variants within each fitness category were further clustered into two subgroups, according to whether the residue substitution corresponded to a conservative (C) or non-conservative (NC) replacement of the native amino acid, and mean residual scores were computed for each subgroup of mutants (Fig. 2, gray and white bar graphs, respectively). By clustering the 20 amino acid types into six subsets as [(A, S, T, G, P), (D, E, N, Q), (R, K, H), (F, Y, W), (V, L, I, M), (C)] based on their physicochemical similarities, intra-class residue replacements are conservative while interclass substitutions are considered to be non-conservative (Dayhoff et al., 1978). According to Fig. 2, the Ras variant subgroups defined by non-conservative residue replacements within each fitness category drive the overall structure-function relationship, while the Ras variants based on conservative residue substitutions form two subgroups that minimally impact structure (i.e., their mean residual scores are 0.02 and -0.05) regardless of the functional consequence (i.e., increased or decreased fitness category, respectively).

Alternatively, distribution of the Ras variants revealed that among the 1567 variants belonging to the increased fitness category, 905 had residual scores ≥0 while 662 had residual scores <0; similarly, among the 1568 variants in the decreased fitness class, 692 had residual scores ≥0 while 876 had residual scores <0. A chi-square test applied to the data led to rejection of the null hypothesis that no association exists between fitness level and residual scores (χ^2^ = 58.2, 1 degree of freedom; *p* < 0.0001).

### 3.2 Residual scores distinguish between categories of Ras amino acids

**Fig. 3:**
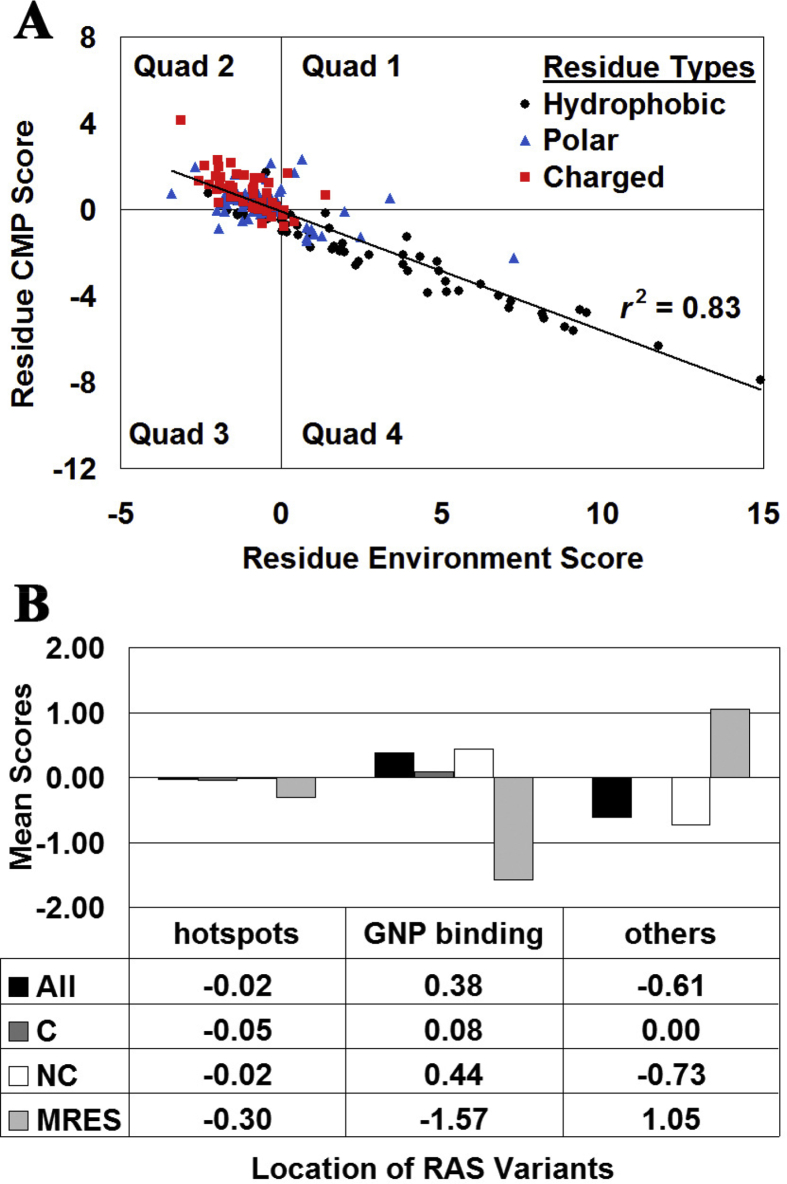
(A) CMP scores for the Ras amino acids are strongly inversely correlated with their residue environment scores. Furthermore, the plot clusters the amino acids according to their polarity. (B) Specific groups of Ras residues are distinguished from one another with the computational mutagenesis data. Hotspots are Ras positions at which nearly all residue substitutions lead to gain-of-function (i.e., increased fitness) variants, and GNP is a slowly hydrolyzing analogue of GTP. All refers to the mean of the residual scores for all Ras variants at all positions belonging to each group, and C/NC are the subsets of all variants in each group that represent conservative/non-conservative amino acid substitutions. MRES refers to the mean of the residue environment scores for all residue positions in each group. The apparent inverse relationship between the mean of the residual scores for all Ras variants in a group (All) and MRES is a consequence of the inverse correlation in (A).

Prior to implementing the computational mutagenesis, a residue environment score (RES) was computed for each of positions 1–166 in the native Ras protein structure from its Delaunay tessellation. At each position, a comprehensive mutational profile (CMP) score was also calculated as the mean of the residual scores for the set of Ras variants corresponding to all 19 amino acid substitutions of the native residue. A strong inverse correlation (*r*^*2*^ = 0.83) was observed between CMP and RES scores over the 166 Ras protein positions (Fig. 3A). At each position, the 19 Ras variants were further segregated into either conservative (C) or non-conservative (NC) substitutions of the native amino acid, and the residual scores of the variants in each subgroup were averaged to yield separate C-CMP and NC-CMP scores. These data revealed NC substitutions (*r*^*2*^ = 0.82) to be the driving force behind the overall correlation in Fig. 3A, with minimal contribution from C substitutions (*r*^*2*^ = 0.15). The 166 residue positions of Ras plotted in Fig. 3A appear to cluster according to polarity of the native amino acids, with most hydrophobic/apolar and charged residues residing in Quadrants 4 and 2, respectively, while polar residues form a diffuse pattern around the origin. Moreover, a chi-square test applied to the distribution of Ras residues over the (4 Quads) × (3 polarities) as depicted in Fig. 3A led to rejection of the null hypothesis that no association exists between native residue polarities and quadrant locations (χ^2^ = 74.4, 6 degrees of freedom; *p* < 0.0001).

Prior experimental studies identified structural or functional roles for certain amino acid positions in Ras, including those that participate in GTP binding (Pai et al., 1990), as well as hotspot positions (Bandaru et al., 2017) at which most if not all residue replacements yield gain-of-function (i.e., increased fitness) Ras variants. These two annotated groups of amino acid positions in Ras were analyzed computationally, first by averaging the RES scores over the positions in each group (mean RES, or MRES, Fig. 3B), and then by averaging the residual scores over all 19*N* Ras variants for all *N* positions in each group (All, Fig. 3B). Within each group, the Ras variants were further segregated into conservative (C) and non-conservative (NC) substitutions of the native amino acid at the given position, and residual scores were similarly averaged over these subgroups. As shown in Fig. 3B, these collective mean scores are effective for distinguishing between Ras amino acid positions grouped by their roles in the protein.

### 3.3 Machine learning models for predicting Ras variant fitness

As previously detailed in the Materials and methods, two distinct types of Ras variant training sets were utilized with the machine learning algorithms. In the first data set, each of the 3135 Ras variants was represented as a 170-dimensional (170D) feature vector whose input attribute components included the mutant residual profile. For the second data set, each variant was represented by a 28D feature vector whose input attributes characterized the local environment of the position mutated in Ras, including components from the residual profile corresponding to the mutated position and its six nearest structural neighbors. However, Ras positions G48 and Q165 possessed only five neighbors each in the structure tessellation; thus, 38 Ras variants defined by the 19 residue replacements at each of those positions were excluded, leaving 3097 Ras variants for the second data set. The output attribute (i.e., variant fitness) in each case was provided either numerically (tree regression) or categorically (random forest classification). Default parameters in the Weka software were used for implementing the machine learning algorithms, with the following exceptions: we used 50 trees with random forest classification, and we applied a bagging meta-learner with tree regression to minimize variance. The bagging procedure utilized ten regression trees generated from bootstrapped data sets, and overall predictions were calculated as the average of those obtained over the ten trees. Lastly, prior to implementing tree regression, we ran the data sets through a nominal-to-binary filter followed by an attribute selection filter (evaluator: CfsSubsetEval; search method: BestFirst), both available with the Weka software.

**Fig. 4:**
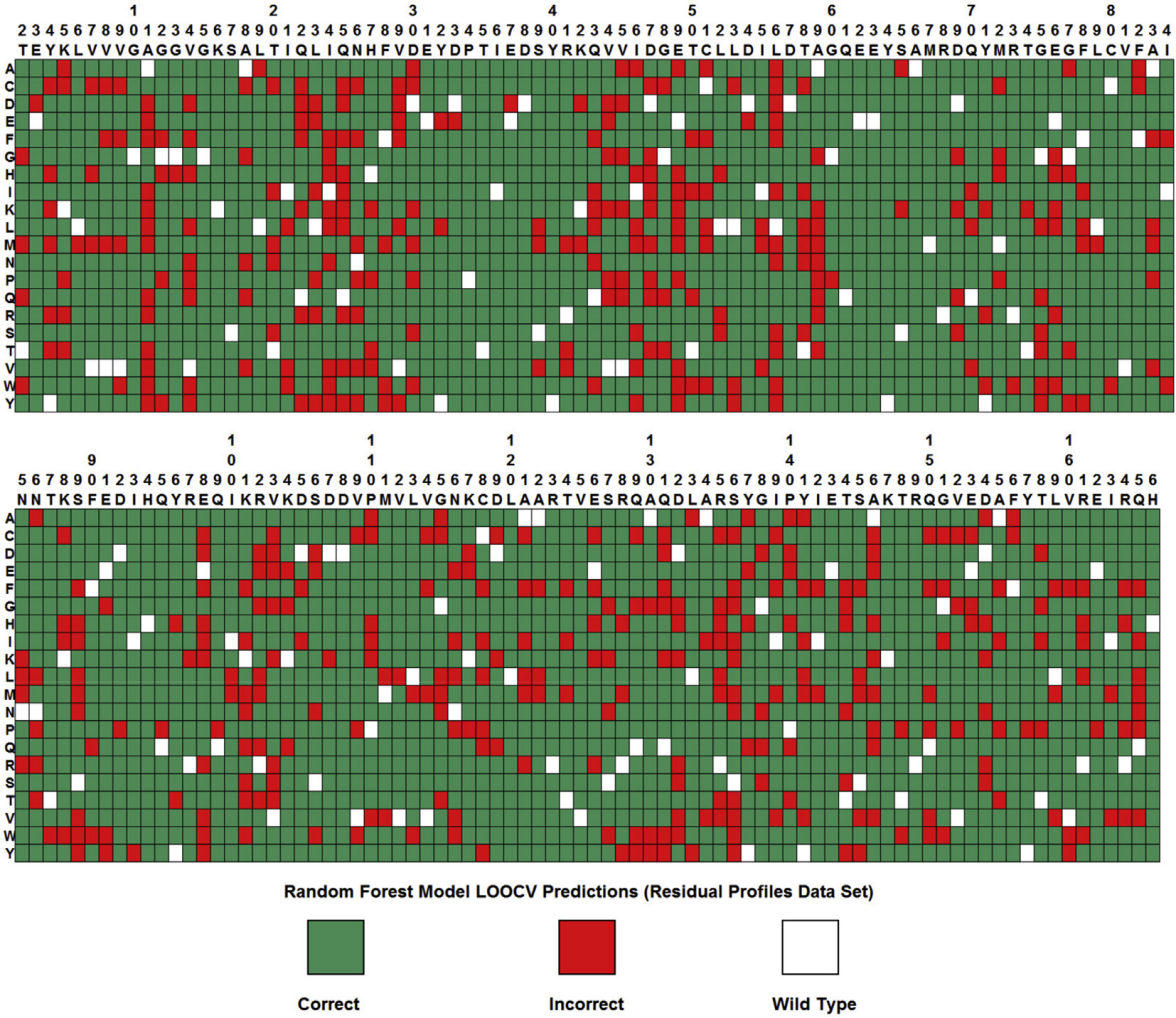
Random forest classification leave-one-out cross-validation (LOOCV) prediction array for all 3135 Ras variants (Residual Profiles data set). Collectively, these predictions yield the performance summary data in the top row of Table 1. Columns correspond to the Ras amino acid positions, and rows represent the 20 different types of residue replacements. A Ras variant is labeled correct (green) if its experimental and predicted fitness categories are identical; otherwise, the variant is labeled incorrect (red).

**Fig. 5:**
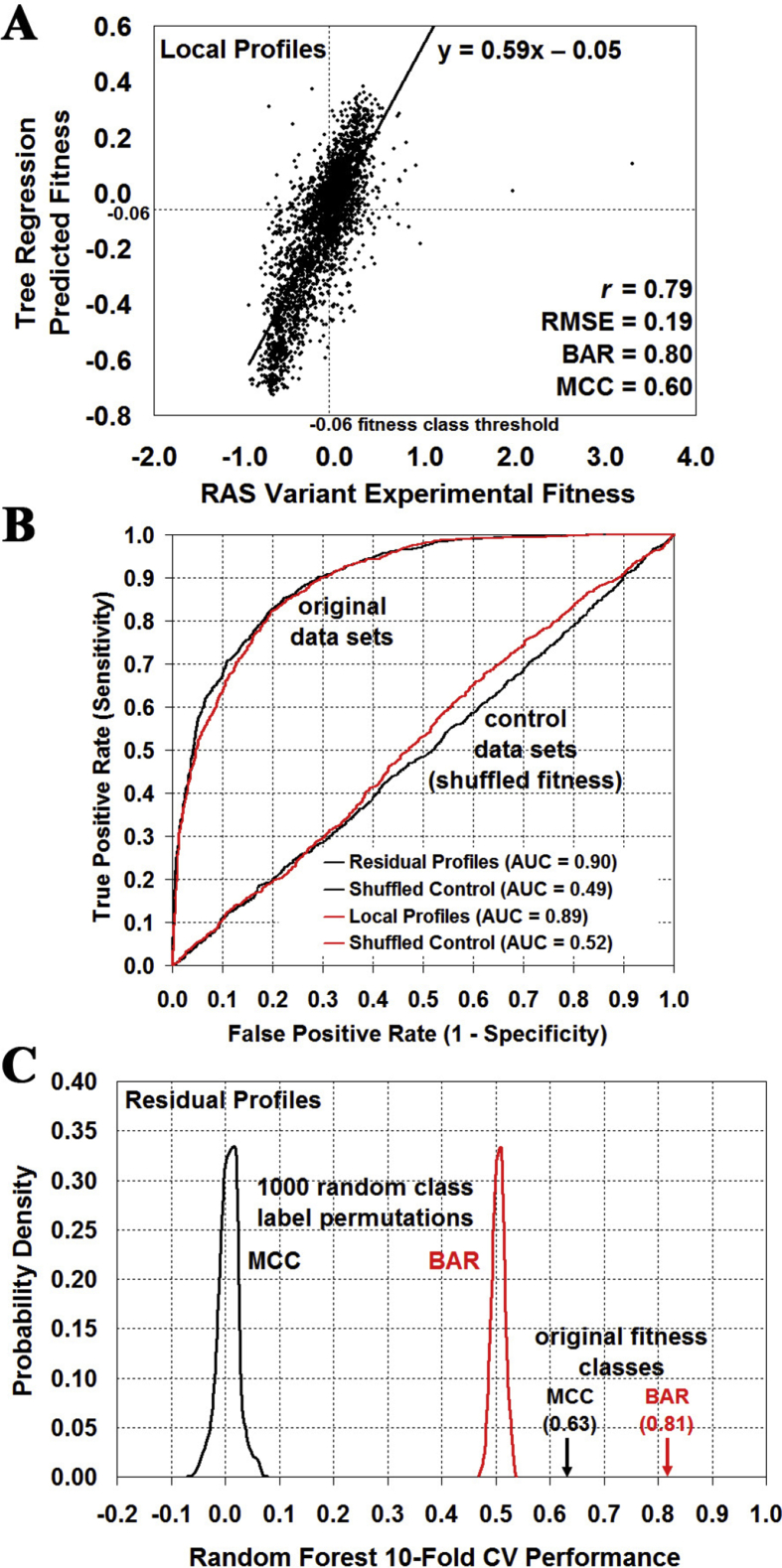
Evaluating the significance of Ras prediction performance. (A) Scatter plot comparing tree regression leave-one-out cross-validation (LOOCV) predictions obtained for the Ras variant fitness values (using the Local Profiles data set) with their experimentally measured values. (B) Random forest LOOCV ROC curves obtained by using the original Residual Profiles and Local Profiles data sets of Ras variants, as well as a control set for each generated by a single random shuffling of the Ras variant fitness classes among one another. (C) Distribution of random forest 10-fold CV prediction performance over 1,000 random fitness class label permutations, compared with results using the original data set (BAR, balanced accuracy rate; MCC, Matthew's correlation coefficient).

Leave-one-out cross-validation (LOOCV) performance results are presented in Table 1 (Residual Profiles = 170D feature vectors, Local Profiles = 28D feature vectors), as are analogous results using control data sets generated by randomly shuffling the fitness output attributes (values or classes) for the Ras variants in each original set among one another. The array presented in Fig. 4 explicitly identifies the random forest LOOCV predictions obtained for all 3135 Ras variants by using the Residual Profiles data set. Results presented in Table 1 for the shuffled control data sets starkly highlight the significance of signals encoded in the Ras variant input attributes for effectively determining their fitness values (regression), as illustrated in Fig. 5A, and for accurately distinguishing fitness categories (classification); moreover, in the latter case of classification, impressive AUC values reported using the original data sets display dramatic drops with the randomly shuffled control data sets to levels near 0.5, which is indicative of models capable of performing no better than random guessing (Fig. 5B).

**Table 1:** Leave-one-out cross-validation (LOOCV) performance on RAS variant data sets.

Random Forest Classification	Se	Sp	PPV	BAR	MCC	AUC
Residual Profiles	0.85	0.78	0.79	0.82	0.63	0.90
Residual Profiles (Shuffled Classes)	0.49	0.49	0.49	0.49	−0.02	0.49
Local Profiles	0.83	0.79	0.80	0.81	0.61	0.89
Local Profiles (Shuffled Classes)	0.51	0.52	0.52	0.52	0.03	0.52

Tree Regression	*r*	RMSE		BAR	MCC	

Residual Profiles	0.75	0.20		0.77	0.55	
Residual Profiles (Shuffled Values)	−0.02	0.31		0.49	−0.01	
Local Profiles	0.79	0.19		0.80	0.60	
Local Profiles (Shuffled Values)	0.01	0.31		0.51	0.02

Subsequently we performed a more systematic assessment of statistical significance by applying 10-fold CV and evaluating performance over each original data set as well as over 1000 control sets obtained by randomly shuffling the fitness output attributes among the Ras variants in each of the original sets. Implementing random forest classification with the original Residual Profiles data set yielded performance measures of BAR = 0.81 and MCC = 0.63, while the collective results over all 1000 shuffled control data sets led to BAR and MCC values that were distributed within narrow windows centered around values consistent with random guessing (BAR = 0.00 ± 0.02 and MCC = 0.50 ± 0.01), so the *p*-value for predictive power of the model is less than 0.001 (Fig. 5C). Similar results were obtained by comparing the performance of the original Local Profiles data set (BAR = 0.81 and MCC = 0.62) with the distribution of the same metrics over 1000 shuffled controls (BAR = 0.00 ± 0.02 and MCC = 0.50 ± 0.01). Implementing tree regression and applying 10-fold CV on the original data sets as well as 1000 shuffled controls proved to be equally significant: Residual Profiles (original: *r* = 0.75; shuffled: *r* = 0.00 ± 0.02); Local Profiles (original: *r* = 0.78; shuffled: *r* = 0.00 ± 0.02).

To benchmark the approach described in this study, we compared predictions obtained using our random forest model (Residual Profiles data set) with seven related state-of-the-art methods (Table 2): STRUM (Quan et al., 2016), MutationAssessor (Reva et al., 2011), AUTO-MUTE (Masso and Vaisman, 2010a, 2014), PMut (Lopez-Ferrando et al., 2017), CUPSAT (Parthiban et al., 2006), PROVEAN (Choi and Chan, 2015), and SNAP (Bromberg and Rost, 2007). Two-thirds of the 3135 Ras variants were randomly selected for training our random forest model, and the remaining 1053 Ras variants were held-out as a test set and were predicted by our trained model as well as by all related methods. Since MutationAssessor would only provide predictions for 342 of these 1053 variants, prediction performance results were tabulated for all methods based on both the larger and smaller test sets. Results in Table 2 reinforce the capacity of the computational mutagenesis technique to encode signals in the residual profiles of Ras variants capable of effectively discriminating between their respective fitness categories.

**Table 2:** Prediction performance of complementary methods on independent test sets of RAS mutants. We trained an RF model using 66% of mutants, and the remaining 1053 provided a test set. Mutation Assessor could only predict 342/1053 RAS mutants.

Method	1053 Mutants		342 Mutants	
	BAR	MCC	BAR	MCC
Random Forest (this study, Residual Profiles)	0.82	0.64	0.81	0.61
STRUM (Quan et al.)	0.64	0.28	0.64	0.27
MutationAssessor (Reva et al.)	Not Obtained		0.63	0.25
AUTO-MUTE (Masso and Vaisman)	0.61	0.27	0.59	0.26
PMut (Lopez-Ferrando et al.)	0.48	−0.05	0.48	−0.04
CUPSAT (Parthiban et al.)	0.48	−0.04	0.47	−0.08
PROVEAN (Choi and Chan)	0.44	−0.19	0.44	−0.17
SNAP (Bromberg and Rost)	0.44	−0.20	0.42	−0.22

**Fig. 6:**
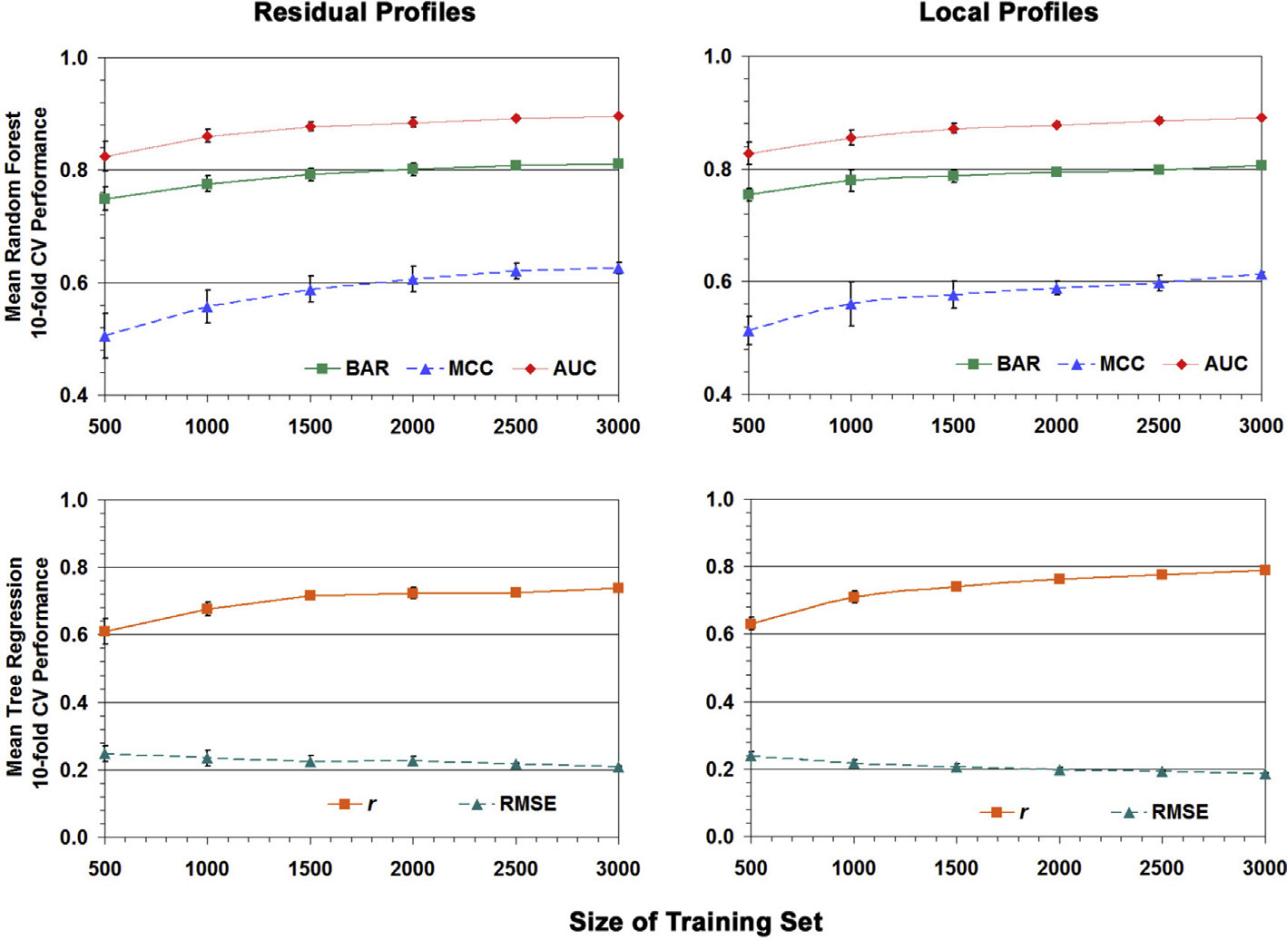
Learning curves. The plots reveal the degree to which performance is improved as the number of Ras variants in the training set is increased. Each point represents the average over ten runs of 10-fold CV, and the error bars indicate the standard deviation. Plots were generated by using both types of Ras variant data sets (Residual Profiles and Local Profiles feature vectors) with both random forest classification and tree regression.

Next, we generated learning curves in order to assess how model performance is affected by the sizes of the Ras variant training sets. The following iterative procedure was implemented separately using each original Ras variant data set (3135 variants in the Residual Profiles set and 3097 variants in the Local Profiles set) and each machine learning algorithm (random forest classification and tree regression). Initially ten subsets each consisting of 500 Ras variants were randomly selected from among all the Ras variants in the original data set, 10-fold CV performance results were obtained for each subset (either BAR, MCC, and AUC with random forest classification, or *r* and RMSE with tree regression), and mean values and respective standard deviations were calculated and plotted (Fig. 6) for these performance measures over the ten subsets. The number of variants randomly selected for each of the ten subsets was iteratively increased by 500, and the procedure concluded by using ten subsets each consisting of 3000 Ras variants. The learning curves produced reflect performance measures that tend to plateau and suggest that perhaps a data set consisting of only 60–65% of the Ras variants are needed to train effective models. In fact, to generate a data set of this size it suffices to consider only the Ras variants defined by using the same 13 amino acids (A, C, E, F, G, H, K, L, P, Q, R, S, and Y) as residue replacements at each position, resulting in either 12 or 13 variants at each Ras protein position, because the remaining 7 amino acids each share similar physicochemical characteristics with one of the 13 residues selected. Removing these 1097 Ras variants leaves precisely 100 × (3135–1097)/3135 = 65% of the data set; moreover, for the random forest LOOCV predictions corresponding to the top row of Table 1 and the array in Fig. 5, a recalculation after removing these 1097 variants proportionally reduces the number of correct and incorrect predictions and again yields BAR = 0.82, which is identical to what was originally obtained with all 3135 variants.

The strict threshold we used to create two Ras variant fitness categories generally makes predictions more challenging for variants as their corresponding fitness values approach that threshold. In order to assess these effects, we ordered the Ras variants by their fitness values and formed a new data set by selecting 500 Ras variants from each of the two opposite extremes of the threshold (i.e., those with the most positive and negative fitness values), for a total of 1000 variants belonging to two equal-sized fitness categories (i.e., increased and decreased). Random forest 10-fold CV predictions, with these Ras variants represented using their 170D vectors as in the Residual Profiles data set, yielded a substantial increase in performance with BAR = 0.93, MCC = 0.86, and AUC = 0.98. This dataset included a total of 47 variants occurring at positions G12, G13, Q61, and K117 known to be mutated in human cancer where they display strong gain-of function (i.e., increased fitness), and 38/47 (81%) of these variants were correctly predicted. We repeated this exercise by selecting 750 Ras variants from each of the two opposite extremes of the threshold, to generate a data set consisting of 1500 variants belonging to two equal-sized fitness categories. Random forest 10-fold CV predictions with this enlarged set led to BAR = 0.92, MCC = 0.84, and AUC = 0.98. For this data set, a total of 58 Ras variants occur at the four positions mutated in human cancers, and 52/58 (90%) of them were correctly predicted. For such sets of Ras variants at opposite extremes of the fitness scale, these combined observations reinforce the observation that signals encoded in the Ras variant residual profiles enable accurate predictions, since models learned with fewer data typically display reduced prediction performance.

As a final task, we explored the accuracy of random forest LOOCV predictions with the Local Profiles data set based on the polarities of the native and replacement residues defining each Ras variant, as well as on the depth and secondary structure of the mutated position in the Ras protein structure. Table 3 reveals that Ras variants for which charged amino acids replaced polar (i.e., uncharged) or apolar residues were predicted most accurately, while predictions for polar to apolar substitutions were the least accurate. For this analysis, residues were categorized as follows: polar (C, G, H, N, Q, S, T, W, Y); apolar (A, F, I, L, M, P, V); and charged (D, E, K, R). Lastly, predictions for mutations at residue positions on the protein surface were found to be the least accurate relative to those that are more buried, which is consistent with the fact that predictions for mutations at residue positions in Ras helices were least accurate when compared to strands and coils (Table 4).

**Table 3:** Mean random forest leave-one-out cross-validation (LOOCV) prediction performance (Ras variant Local Profiles) based on side chain polarities of the native and new amino acids at the mutated position.

∗new/native	Polar			Apolar			Charged		
	BAR	MCC	%	BAR	MCC	%	BAR	MCC	%
Polar	0.79	0.58	15	0.72	0.47	13	0.85	0.70	8
Apolar	0.79	0.59	17	0.76	0.52	11	0.84	0.66	8
Charged	0.82	0.65	13	0.81	0.63	10	0.81	0.61	5

∗ Explicit distribution of residues among these categories is provided in the text.

**Table 4:** Mean random forest leave-one-out cross-validation (LOOCV) prediction performance (Ras variant Local Profiles) based on depth and secondary structure.

	BAR	MCC	%
Depth			
Buried	0.80	0.60	50
Undersurface	0.84	0.68	24
Surface	0.74	0.50	26
Secondary Structure			
Strand	0.80	0.60	27
Helix	0.77	0.54	37
Coil	0.81	0.63	36

## 4 Conclusion

With access to coordinate data for a native 3D structure of a human oncogenic protein, this study details a complementary approach for modeling its collection of variants and predicting their functional consequences. The computational mutagenesis methodology employed here generates a residual profile vector for each variant, one that characterizes the structural impact that the mutation has on the protein. An inherent structure-function relationship in proteins implies that every such vector for a variant has the capacity to distinguish the corresponding functional impact. Machine learning algorithms require seed training data in order to generate predictive models, and suggestions were proposed earlier for the number and distribution of variants that would perhaps be needed to train a reliable model. These variants would need to be experimentally synthesized and the functional impacts (e.g., fitness) of the mutations assessed. Fortunately, significant advances have been made recently with, for example, deep mutational scanning techniques that are transforming the study of proteins, by coupling large-scale synthesis of protein variants with experimental determination of their functional consequences. In this regard, collaborations with experimentalists skilled at performing deep mutational scans and obtaining fitness data for protein variants would serve to facilitate additional studies on the related human K-Ras and N-Ras isoforms, as well as on a wide array of proto-oncogenes for which high-resolution 3D protein structures have been determined.

## Declarations

### Author contribution statement

Majid Masso: Conceived and designed the experiments; Performed the experiments; Analyzed and interpreted the data; Contributed reagents, materials, analysis tools or data; Wrote the paper.

Arnav Bansal, Preethi Prem, Akhil Gajjala: Performed the experiments; Analyzed and interpreted the data.

Iosif I. Vaisman: Conceived and designed the experiments.

### Funding statement

This research did not receive any specific grant from funding agencies in the public, commercial, or not-for-profit sectors.

### Competing interest statement

The authors declare no conflict of interest.

### Additional information

No additional information is available for this paper.
